# Association of dopamine receptor polymorphisms with schizophrenia and antipsychotic response in a South Indian population

**DOI:** 10.1186/1744-9081-3-34

**Published:** 2007-07-25

**Authors:** Neetha N Vijayan, Sujatha Bhaskaran, Linda V Koshy, Chandrasekhar Natarajan, Lekshmy Srinivas, Chandrasekharan M Nair, Priya M Allencherry, Moinak Banerjee

**Affiliations:** 1 Human Molecular Genetics Laboratory, Rajiv Gandhi Centre for Biotechnology, Thiruvananthapuram, Kerala, India; 2 Nair's Hospital, Ernakulam, Kerala, India; 3 Mental Health Centre, Kerala, India

## Abstract

**Background:**

Alterations in the dopamine transmission and receptor density are hypothesized in the pathophysiology of schizophrenia but ethnic disparities are reported to exist in disease association and therapeutic response to psychotropic medication. Antipsychotics have higher binding affinity to D2 subtype of dopamine receptor. DRD2 Cys311, TaqIB1 and TaqIA1 variants are considered to have either reduced affinity for dopamine and hypo-dopaminergic activity.

**Methods:**

We examined the role of Taq1B, Taq1D, S311C, H313H and Taq1A polymorphisms of DRD2 gene in schizophrenia and antipsychotic treatment response in 213 patients and 196 controls from a homogenous South Indian population. A more detailed genotype phenotype association analysis was carried out to understand the disease in terms of its socio-cultural factors.

**Results:**

H313HTT genotype was found to be associated with schizophrenia (P = 0.004) while TaqIB1B1 genotype was significantly associated with higher psychopathology score. When treatment response was considered H313HCC, TaqIA2A2 and Taq1D1D1 had higher mean improvement scores. TaqID1D1 and H313HTT genotype were found to be significantly higher in responders than in nonresponder group. Distinct shift in the LD patterns of responder and non-responder group was observed. Certain symptoms were characteristic of our patient population. Following medication the scores and presentation of these symptoms tend to vary in the responder and non-responder groups.

**Conclusion:**

Based on genotype phenotype correlations it can be suggested that certain polymorphisms can be defined for their critical functions in disease and their role in treatment response in South Indian population. The present study suggests that in addition to ethnic bias, socio-cultural factors should also be considered while evaluating genotype phenotype correlations, in association and treatment response to complex disorders like schizophrenia.

## Background

Schizophrenia is a common but complex disorder, with no consensus on the prevalence rate of this disease in India. There appears to be no clear pockets of high or low prevalence of schizophrenia in Indian population [1]. On the other hand, the incidences of the factors that associate with schizophrenia such as, economic status, migration [2] and suicidal tendencies are three to four fold higher in the state of Kerala, South India, than the national average.

Family, twin and adoption studies suggest that genetic factors play a significant role in the etiology of schizophrenia [3]. Alterations in the dopamine transmission and receptor density have been hypothesized in the pathophysiology of schizophrenia [4]. Pharmacological observations reveal that excess dopaminergic activity lead to the psychotic symptoms of this disorder [5-7]. Several studies have shown positive association of D2 subtype of dopamine receptor (DRD2) in schizophrenia [8-12] however, this was refuted by many others [13-16]. Therefore, it has been suggested that population gene frequencies have to be considered when interpreting the association between an allele and a disease because there is a chance that association might be due to a stratified ethnic background rather than a direct causal relation. Similarly, ethnic disparities have also been observed in response to psychotropic medication. Antipsychotics have higher binding affinity to D2 subtype of dopamine receptor (DRD2) in schizophrenic patients [17]. This binding affinity is strongly correlated with the efficacy of the drugs in controlling the positive symptoms of schizophrenia [18]. Therefore mutations in DRD2 receptor might have variable effect on the presentation of symptom and treatment response. DRD2 has 8 exons and several of the SNPs in the DRD2 gene have been screened for association with the disease. Among the three non-silent mutations DRD2 V96A, P310S, S311C in the exonic region of DRD2, S311C has been reported to have significant association in several world populations [19]. Earlier studies have shown that Cys311 variant of DRD2 has half the affinity for dopamine in comparison to its wild type variant [20]. DRD2 Taq1A and Taq1B polymorphisms support hypo-dopaminergic activity. *In vitro *and *in vivo *studies suggest that Taq1A1 and Taq1B1 alleles have reduced D2 density in the striatum [21,22]. In contrast to association studies very few studies have evaluated the role of DRD2 receptor variants in treatment response. Most often the association or treatment response studies have been conducted in relation to schizophrenia and not in identifying the socio-cultural factors that associate with schizophrenia in presenting various symptoms.

The aim of the present study was to investigate the involvement of various alleles, genotypes, haplotypes and their linkage disequilibrium status of Taq1B (rs 1079597), Taq1D (rs1800498), S311C (rs1801028), H313H (rs 6275), and Taq1A (rs 1800497) polymorphisms in DRD2 receptor gene in causing schizophrenia and their relation to treatment response in a South Indian population of Kerala. The study was further aimed at understanding the socio-cultural factors that associate with schizophrenia in presenting various symptoms and their genotype phenotype correlations.

## Methods

### Study design

Subjects included 213 schizophrenic patients (81 Males and 132 Females) with mean age 34.40 ± 10.86 of South Indian state, Kerala. All the patients were diagnosed as per the DSM-IV criteria [23] for mental disorders. Complete patient history, their medication and follow up of all the patients were maintained for independent review. Two independent psychiatrists and a clinical pharmacologist further assessed the patient history, diagnosis and medication of these patients. Baseline rating of the patients based on Extended Brief Psychiatric Rating Scale (BPRS-E, 24 item, score 1–7) [24] was done at the time of first consultation. Medication profile was monitored for all the follow up periods. The study was set in its natural background, therefore, the patients were not controlled for their medication with antipsychotics. Majority of the patients had been given both typical and atypical antipsychotics as per their medical requirements. The most frequently administered drugs were clozapine, haloperidol and risperidone. The chlorpromazine equivalents ranged from 423.8 ± 253.9 mg per day. One-year follow up was considered for evaluating the treatment response. Clinical improvement was assessed by percent change scores between initial baseline BPRS score and BPRS score after one-year follow-up [25]. Out of the 213 patients, 181 patients completed the one year follow-up. Patients were classified into responders and non-responders by standard one scale criteria for evaluating response to antipsychotics. In standard one-scale criteria the patients with improvement of 50% or more in their BPRS ratings after one year follow up were classified as "responders" and others as "non responders". 122 patients met the criteria of "responders" and 59 as "nonresponders" to antipsychotic treatments. Control samples included 196 ethnically and age matched random individuals who had no family history of schizophrenia and had never taken any neuroleptics in their lifetime. For understanding the socio-cultural factors each individual symptom was assessed for their overall representation (frequency) in the disease population while the psychopathology (severity) of symptoms was assessed by the BPRS score of each individual symptom. Frequency was represented by presence or absence of each individual symptom presented in the patient population without considering the BPRS scores while severity was represented based on the BPRS rating score of each individual symptom in the patient population. Similar evaluation of frequency and severity of symptom was done in the responder and non responder patient groups too.

### Genotyping

Peripheral blood was collected from all the individuals in EDTA coated vials. The study was approved by Institutional Ethics Committee for biomedical subjects as per the ICMR guidelines. Genomic DNA was isolated from lymphocytes according to Sambrook and Russell [26]. DRD2 SNPs were screened as described earlier for S311C [27], H313H [28], TaqIA [29], TaqIB and TaqID [30]. PCR was carried out in a total volume of 25 μl containing 100 ng DNA, 200 μM dNTP, 1 μM of each primer, 1X buffer (NEB, Inc., USA) and 0.5 units of Taq polymerase (NEB, Inc., USA). PCR was programmed for initial denaturation at 94°C for 3 min followed by 35 cycles of denaturation at 94°C for 30 s, annealing for 45 s (depending on the primer), extension at 72°C for 1 min and a final extension of 72°C for 5 min in a Eppendorf Mastercycler (Table 1). PCR products were digested with restriction enzymes (depending on the nature of polymorphism) according to manufacturer's protocol (NEB, Inc., USA), separated on 3% agarose gel and stained with ethidium bromide for visualisation.

**Table 1:** PCR primers, annealing temperatures for PCR and the restriction enzymes used to detect different DRD2 polymorphisms.

**Polymorphism**	**Primers**	**Annealing temp.**	**Restriction Enzyme**
Taq1B	F 5'GATACCCACTTCAGGAAGTC 3' R 5'GATGTGTAGGAATTAGCCAGG 3'	60°C	Taq1A
Taq1D	F 5'CCCAGCAGGGAGAGGGAGTA 3' R 5'GACAAGTACTTGGTAAGCATG 3'	60°C	Taq1A
S311C	F 5'TTGGGCATGGTCTGGATCTCAAA 3' R 5'CCAGCTGACTCTCCCCGACCGGT 3'	65°C	Sau961
H313H	F 5'ATCCTGCAGCCATGG 3' R 5'ATTGTCCGGCTTTACC 3'	55°C	Nco1
Taq1A	F 5'CCCTTCCTGAGTGTCATCA 3' R 5'CGGCTGGCCAAGTTGTCTA 3'	58°C	Taq1A

### Statistical analysis

Allele frequency, genotype frequency, Hardy-Weinberg's equilibrium, linkage disequilibrium (LD) and haplotype frequencies were calculated with the software COCAPHASE [31]. LD was analyzed and plotted using Haploview [32]. Statistical comparison of clinical variable and categorical data between the patients and controls were analyzed using Chi-square (χ^2^) test. Fisher's exact t-test was used whenever necessary. A "P" value of <0.05 was considered to be statistically significant. Odds ratio (OR) and confidence interval was also calculated wherever required. To reveal the basic structure of the findings, the baseline characteristics were analyzed. Mann Whitney U test was used to understand the relation between genotypes and severity of each individual symptom at initial presentation and after one year follow-up. This was done by the SPSS version 11 software program.

## Results

Demographic features of the subjects are given in Table 2. No significant difference was observed in the age of onset between the genders. No preference of gender and disease was observed. Baseline BPRS scores and percent improvement score also did not vary between the genders. The allele and genotype frequencies of DRD2 SNPs are shown in Table 3. None of the SNPs show deviation from Hardy-Weinberg's equilibrium in the patient and control population.

**Table 2:** Demographic characteristics of patient population.

**Sex (n)**	**Mean Age**	**Mean Age of onset**	**BPRS Baseline Score**	**Final BPRS Sore**	**Mean % change in BPRS Score**
F (132)	33.95 ± 12.9	24.10 ± 8.73	62.23 ± 11.59	39.30 ± 10.10	61.87 ± 23.38
M (81)	33.36 ± 8.9	24.95 ± 6.77	59.11 ± 9.58	37.91 ± 9.108	58.50 ± 30.62

**Table 3:** Allele and genotype frequencies of dopamine receptor polymorphisms in patient and control groups.

**Locus**	**Genotype Frequency**			**χ**^**2**^	***P***	**Allele Frequency**		**χ**^**2**^	***P***
**Taq1B (rs 1079597)**	**B1B1**	**B1B2**	**B2B2**	**2.0**	**0.36**	**B1**	**B2**	**0.03**	**0.86**
Cases (n = 212)	0.05(11)	0.41(86)	0.54(115)			0.25(108)	0.75(316)		
Controls (n = 196)	0.08(16)	0.36(70)	0.56(110)			0.26(102)	0.74(290)		
**Taq1D (rs 1800498)**	**D1D1**	**D1D2**	**D2D2**	**2.49**	**0.29**	**D1**	**D2**	**0.004**	**0.95**
Cases (n = 211)	0.12(25)	0.58(123)	0.30(63)			0.41(173)	0.59(249)		
Controls (n = 195)	0.15(30)	0.51(99)	0.34(66)			0.41(159)	0.59(231)		
**S311C (rs 1801028)**	**GG**	**GC**	**CC**	**0.41**	**0.81**	**G**	**C**	**0.01**	**0.92**
Cases (n = 210)	0.80(169)	0.18(38)	0.10(3)			0.89(376)	0.11(44)		
Controls (n = 195)	0.81(159)	0.16(32)	0.02(4)			0.90(350)	0.10(40)		
**H313H^1 ^(rs 6275)**	**TT**	**TC**	**CC**	**9.60**	0.008*	**T**	**C**	**2.92**	**0.09**
Cases (n = 213)	0.36(76)	0.43(92)	0.21(45)			0.57(244)	0.43(182)		
Controls (n = 193)	0.23(44)	0.57(110)	0.20(39)			0.51(198)	0.49(188)		
**Taq1A^2 ^(rs 1800497)**	**A1A1**	**A1A2**	**A2A2**	**4.88**	**0.09**	**A1**	**A2**	**0.57**	**0.45**
Cases (n = 212)	0.08(17)	0.44(93)	0.48(102)			0.30(127)	0.70(297)		
Controls (n = 194)	0.15(29)	0.40(77)	0.45(88)			0.35(135)	0.65(253)		

^1^H313H dominant model (TT Vs TC+CC) *P *= 0.0045, Odds = 1.879, CI = 1.212 to 2.911

^2 ^Taq1A recessive model (A1A1 Vs A1A2+A2A2) *P *= 0.029, Odds = 0.4960, CI = 0.2632 to 0.9348

### DRD2 association with schizophrenia

**Figure 1:**
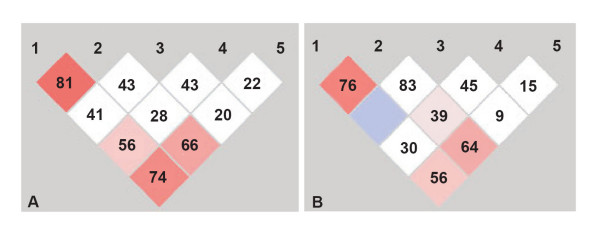
LD patterns of DRD2 SNPs in cases (a) and controls (b).

When the allele frequencies were compared between cases and controls we did not find any significant association with any of the individual SNPs with the disease. Interestingly the homozygous genotype of alleles that represented lower dopaminergic activity or reduced receptor density, were less frequent in our study population. However, when the role of individual genotype frequencies were compared for disease association, DRD2 H313H TT genotype was found to be significantly associated with the disease (P = 0.008, χ^2 ^= 9.60, df = 2). While using a model based approach for disease association a similar significant positive association of H313H with TT genotype in dominant model (TT Vs TC+CC, P = 0.0045), was observed while DRD2 TaqIA with A1A1 genotype in a recessive model (A1A1 Vs A1A2+A2A2, P = 0.029) was observed as a protective genotype for the disease (Table 3). On further analysis with 2 to 5 marker haplotypes, we observed a distinct pattern of haplotypes in cases and controls and several haplotypes were found to be significantly associated (Table 4). Haplotype frequency less than 5% in both the groups were not considered for interpretation. DRD2 H313H with T allele and Taq1A with A2 allele were observed to be common in 2 and 3 locus haplotypic association with the disease. The C allele of H313H seems to be associated with protective haplotype in majority of the haplotypic combinations. Extensive pairwise LD was observed between TaqI "B" and TaqI "D" and between TaqI "B" and TaqI "A in the control group, however, in the same pairwise combinations in the patient group, a shift in the pattern of LD was observed (Fig 1). In the patient group the pairwise LD between the TaqI "B" and TaqI "A" reduced while between TaqI "D" and "S311C"a very distinctive increase was observed.

**Table 4:** Two – Five marker haplotype frequency in patient and control groups.

		**Haplotype frequency**			
				
**Locus**	**Haplotype**	**Cases**	**Controls**	***P***	**Odds**
Locus 4-5	1-2	38.12	30.34	0.024	1.41
Locus 3-4-5	1-1-2	35.5	28	0.029	1.44
Locus 1-2-3-4	1-1-1-2	4.6	0.2	0.019	33.94
Locus 1-2-3-4	2-1-1-2	4.9	11.5	0.012	6.34
Locus 1-2-3-4-5	2-1-1-2-2	3.95	10.72	0.009	6.72

None of the alleles or genotypes was found to be associated with early onset schizophrenia, though a non significant trend was observed for TaqI B2B2 genotype with age of onset below 20 (Data not shown). Patients were further assessed for their extent of psychopathological symptoms based on their BPRS rating at initial presentation. Using Mann Whitney U test for all genotype and overall disease phenotype correlations, we observed that among all genotype comparisons, TaqI B1B1 genotype individuals showed significantly higher (P = 0.017) psychopathology scores (Data not shown).

### DRD2 association with treatment response in schizophrenia

Out of 213 patients the BPRS rating with one year follow up was available for only 181 individuals. Out of 181 patients 100% improvement in BPRS score was observed in only 9 patients. When the common genotypes were compared in these nine patients, we observed that TaqI A2A2 and TaqI B2B2 genotypes were present in 88.87 % of these patients. Average percentage improvement after one year follow up was observed to be 60%. When individual genotypes were compared using Mann-Whitney U test for overall improvement, we observed that H313H CC genotype, TaqI A2A2 and TaqI D1D1 genotype had a significantly higher mean percent improvement when compared to TC genotypes (P = 0.042), A1A2 genotype (P = 0.0487), D1D2 (P = 0.0066) and also D2D2 genotype (P = 0.01) respectively (see Additional file).

**Figure 2:**
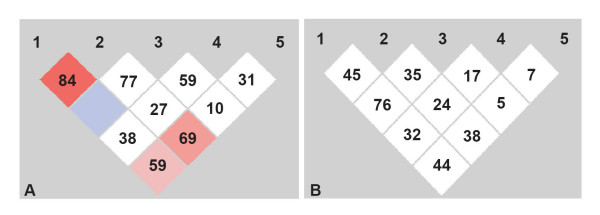
LD patterns of DRD2 SNPs in responders (a) and non responders (b).

The patients were further classified into responder and non responder groups based on 50% improvement criteria. Based on this threshold, 67% of the individuals were observed to fall in the responder group. None of the individual SNPs showed any association with the responder and the non-responder patient group (Table 5). However, while considering a model based approach we observed that DRD2 Taq1D with D1D1 genotype in a recessive model (D1D1 vs D1D2 + D2D2, P = 0.0226) and H313H with TT genotype in a dominant model (TT vs TC + CC, P = 0.049) show significant association with the responder group (Table 5). Interestingly the H313H TT genotype that was significantly associated with the disease was also found to be associated with good response to medication. Patients with poor dopaminergic genotypes 311Cys Cys and TaqI A1A1 were not found to have any relationship with treatment response. The responder and non-responder groups were further screened for 2 to 5 marker haplotypic associations with similar 5% threshold as cut off for interpretation. We found that TaqI B2 and TaqI D1 were significantly high in responders group and were presented frequently in several haplotypic combinations. While TaqI D2 and G allele in S311C polymorphism has been observed to be significantly higher and frequently represented in different haplotypic combinations in the non-responder group (Table 6). Pairwise LD between the SNPs in the responder and non responder patients varied. The LD between TaqI "B" and "D", and TaqI "B" and "A" in the responder group was similar to the overall patient group, while in the non responder group the pairwise LD drastically reduced in all combinations (Fig 2).

**Table 5:** Allele and genotype frequencies of dopamine receptor polymorphism in responder and non responder groups in patient population based on percent response score ≤ 50% ≥.

**Locus**	**Genotype Frequency**			**χ**^**2**^	***P***	**Allele Frequency**		**χ**^**2**^	***P***
**Taq1B (rs 1079597)**	**B1B1**	**B1B2**	**B2B2**	**0.18**	**0.915**	**B1**	**B2**	**0.15**	**0.7**
Responders(n = 121)	0.04(5)	0.42(51)	0.54(65)			0.25(61)	0.751(181)		
Nonresponders(n = 59)	0.05(3)	0.44(26)	0.51(30)			0.27(32)	0.73(86)		
**Taq1D **^1 ^**(rs 1800498)**	**D1D1**	**D1D2**	**D2D2**	**6.14**	0.046*	**D1**	**D2**	**0.65**	**0.42**
Responders (n = 121)	0.15(18)	0.54(65)	0.31(38)			0.42(101)	0.58(141)		
Nonresponders(n = 59)	0.03(2)	0.68(40)	0.29(17)			0.37(44)	0.63(74)		
**S311C (rs 1801028)**	**GG**	**GC**	**CC**	**0.99**	**0.61**	**G**	**C**	**0.26**	**0.61**
Responders (n = 120)	0.79(95)	0.19(23)	0.02(2)			0.89(213)	0.11(27)		
Nonresponders(n = 58)	0.81(47)	0.19(11)	(0)			0.90 (105)	0.10(11)		
**H313H**^2 ^**(rs 6275)**	**TT**	**TC**	**CC**	**5.57**	**0.06**	**T**	**C**	**1.76**	**0.18**
Responders (n = 122)	0.36(44)	0.41(50)	0.23(28)			0.57(138)	0.43(106)		
Nonresponders(n = 59)	0.20(12)	0.58(34)	0.22(13)			0.49(58)	0.51(60)		
**Taq1A (rs 1800497)**	**A1A1**	**A1A2**	**A2A2**	**1.91**	**0.39**	**A1**	**A2**	**1.44**	**0.23**
Responders (n = 121)	0.09(10)	0.42(51)	0.49(60)			0.29(71)	0.71(171)		
Nonresponders(n = 59)	0.08(5)	0.53(31)	0.39(23)			0.35(41)	0.65(74)		

^1^Taq1D recessive model (D1D1vs D1D2 +D2D2) ***P ***= 0.023, Odds = 4.98, CI = 1.115 to 22.25

^2^H313H dominant model (TT Vs TC+CC) ***P ***= 0.0490, Odds = 0.490, CI = 0.2394 to 1.004

**Table 6:** Two – Five marker haplotype frequency in the responder and non responder groups in patient population based on percent response score ≤ 50% ≥.

		**Haplotype frequency**			
				
**Locus**	**Haplotype**	**Responders**	**Nonresponders**	***P***	**Odds**
Locus 4-5	2-1	8.7	18.75	0.0084	2.46
Locus 3-4-5	1-2-1	7.17	16.7	0.0274	4.86
Locus 2-3-4-5	1-1-2-1	2.93	9.73	0.049	3.87

### Evaluation of symptoms

**Figure 3:**
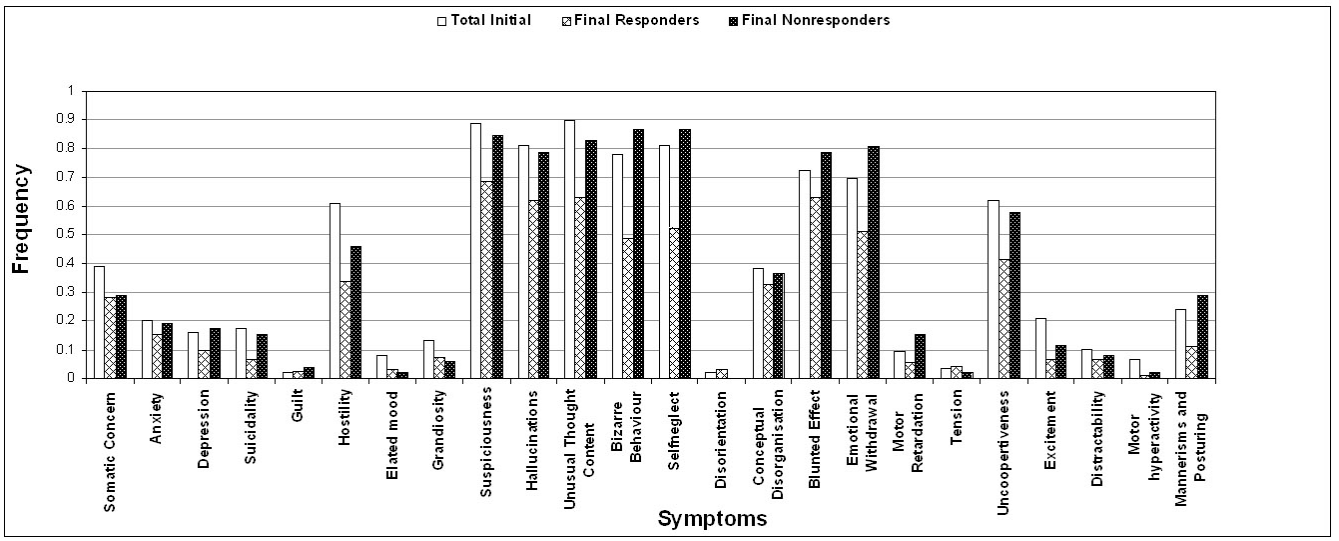
Frequency of symptoms represented at initial presentation compared with presentation of symptoms after one year follow up of medication in responder and non responder patients.

**Figure 4:**
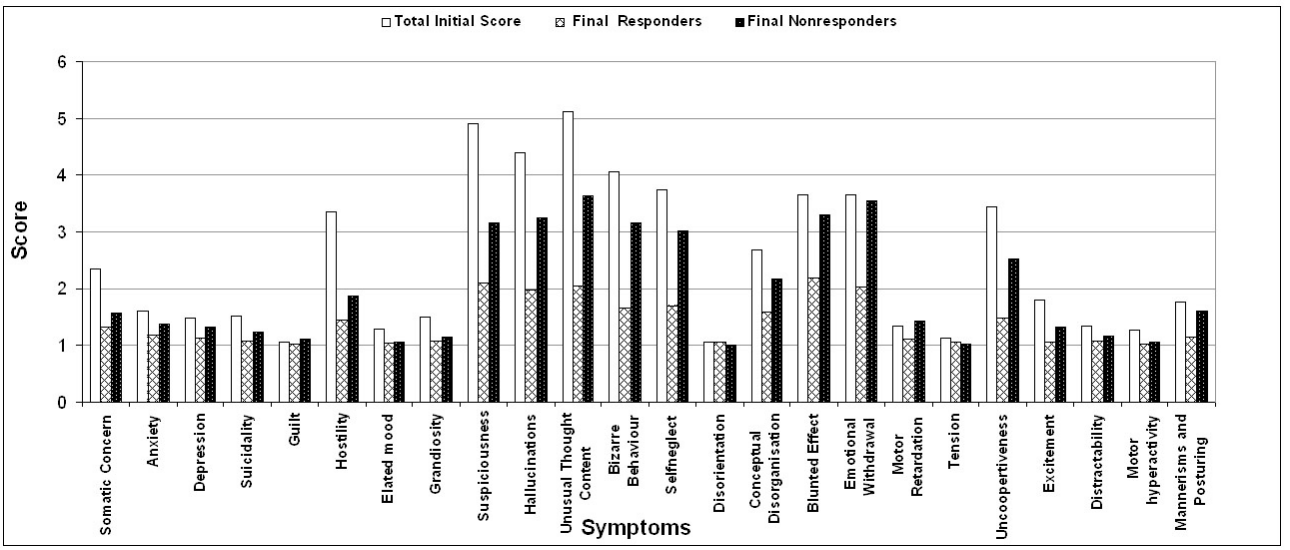
Severity of symptoms based on initial BPRS score compared with the BPRS score after one year follow up of medication in responder and non responder patients.

We further tried to understand the disease in terms of its symptoms, which is believed to be influenced by socio-cultural factors that are associated with South Indian population. Symptoms were assessed for their frequency of presentation and psychopathological scores as described earlier. Representation of individual symptoms above 40% and psychopathology subscores above 3 were only considered for interpretation. Representation of various symptoms in the patient population is shown in Fig 3. Certain positive symptoms such as suspiciousness, hallucinations, bizarre behaviour and hostility and certain negative symptoms such as self neglect, blunted effect, emotional withdrawal and uncooperativeness are the major characteristic symptoms that represented our patient population. Cognitive symptoms such as unusual thought content are also highly represented in our patient population. All these symptoms were represented in more than 60 percent of the patient population. Same set of symptoms were also found to be chronic in presentation when the severity of the symptoms were assessed, however, the severity of the presentation varied (Fig 4).

In a one year follow up assessment, majority of the symptoms were observed to have improved, in the patient population. However, when the same assessment was done in the responder and non-responder patient group, we observed that the representation of symptoms varied in responder and non responder groups. In responder group the representation of certain symptoms such as bizarre behaviour, self neglect, hostility and unusual thought content reduced drastically while a reverse trend was observed in the non-responder group (Fig 3). The symptoms such as bizarre behaviour, self neglect, emotional withdrawal and blunted effect had increased representation while hallucinations, suspiciousness and uncooperativeness display minimal change from initial score in the non responder group. Aggressive symptoms such as hostility seem to have significantly reduced in its representation in both the groups.

Severity of symptoms was also observed to decrease in all the patients. However, when responder and non-responder groups were compared, the severity scores in the responder group were observed to decrease drastically when compared to non-responder group (Fig 4). In the responder group the severity scores decreased by a score of 1.5–2 while in the non responder group it decreased by 1–1.5 in a 0–6 point scale. Based on these thresholds of scores in the responder and non responder groups we found that emotional withdrawal and blunted effect had no improvement in the non-responder group while in responder group symptoms such as blunted effect, self neglect and bizarre behaviour had marginal improvement in their severity scores.

### Evaluation of symptoms and genotype

After understanding the disease in terms of its symptoms, it was important to understand the relationship of genotype to a specific phenotype (symptom). Mann Whitney U test was used to compare the genotypes and their relationship with each individual's initial BPRS scores for each symptom (Table 7). The test was independent of overall presentation of symptom in a particular genotype. Among the positive symptoms, severe hallucination was observed to be significantly associated with Taq1 B1B1 genotype. While among the negative symptoms, severe self neglect and uncooperativeness was significantly associated with H313H TC and TT genotype. DRD2 Taq1 A1A1 and A1A2 genotypes were found to be significantly associated with severe self neglect symptoms in schizophrenic patients.

**Table 7:** Relationship of genotype to a specific symptom in patient population based on BPRS score at initial presentation.

**Symptom**	**Polymorphism**	**n**	**Genotype**		**n**	***P *value**
Hallucination	Taq1B	68	B1B1 Vs B1B2	B1B1	5	0.0032
				B1B2	63	
		83	B1B1 Vs B2B2	B1B1	5	0.0031
				B2B2	78	
Self neglect	His313His	107	CC Vs TC	CC	39	0.019
				TC	68	
	Taq1A	79	A1A1 Vs A2A2	A1A1	12	0.045
				A2A2	67	
		134	A1A2 Vs A2A2	A1A2	67	0.012
				A2A2	67	
Uncooperativeness	His313His	107	CC Vs TC	CC	39	0.04
				TC	68	
		79	CC Vs TT	CC	39	0.001
				TT	40	

Subsequently, we also tried to understand the genotype phenotype relationship in response to medication. It was observed that in response to medication, the Taq1 D1D1 genotype individuals display improved response in positive symptoms like suspiciousness, hallucination and bizarre behavior (Table 8). Individuals heterozygous for DRD2 H313H TC genotype show poor response to medication for hallucination. However, among negative symptoms, uncooperative behavior was observed to be associated with reduced impact of medication in homozygous DRD2 H313H TT genotype individuals. DRD2 Taq1 A2A2 and B2B2 genotype was observed to be associated with improvement of self neglect symptom.

**Table 8:** Relationship of genotype to a specific symptom in patient population based on BPRS score after one year follow-up.

**Symptom**	**Polymorphism**	**n**	**Genotype**		**n**	***P *value**
Suspiciousness	Taq1D1	102	D1D1 Vs D1D2	D1D1	14	0.023
				D1D2	88	
		55	D1D1 Vs D2D2	D1D1	14	0.04
				D2D2	41	
Hallucination	His313His	105	CC Vs TC	CC	38	0.06
				TC	67	
		106	TC Vs TT	TC	67	0.03
				TT	39	
	Taq1D	55	D1D1 Vs D2D2	D1D1	14	0.03
				D2D2	41	
Bizarre Behavior	Taq1D	55	D1D1 Vs D2D2	D1D1	14	0.045
				D2D2	41	
Self neglect	Taq1A	76	A1A1 Vs A2A2	A1A1	11	0.03
				A2A2	65	
		132	A1A2 Vs A2A2	A1A2	67	0.003
				A2A2	65	
Uncooperativeness	His313His	105	CC Vs TC	CC	38	0.02
				TC	67	
		77	CC Vs TT	CC	38	0.0015
				TT	39	

## Discussion

Alterations in dopamine transmission and dopamine receptors particularly the D2 receptor have long been hypothesized in the pathophysiology of schizophrenia [8,17]. In the past, several studies have been carried out to identify the role of DRD2 polymorphism representing variable receptor densities in schizophrenia [8]. Very few studies have been carried out to evaluate the treatment response of antipsychotics in schizophrenia [33,34]. Racial disparities have been observed in associating various DRD2 SNPs in causing the disease and also in evaluating the treatment response. In the present study the role of various DRD2 SNPs in causing the disease and treatment response of antipsychotics in a more homogeneous population of South India were examined. In addition, we also assess the genotype phenotype correlations based on presentation of individual symptoms and their severity in disease manifestation and treatment response. This is probably the first study which tries to dissect the role of DRD2 polymorphisms in schizophrenia to the level of symptoms. Therefore, an exploratory study of this kind will enhance the understanding of critical factors such as ethnicity, social and cultural events involved in assessing the disease and treatment response.

### Association of DRD2 variants in schizophrenia

In the present study we could find a strong association of DRD2 H313H TT genotype with the disease. This polymorphism has not been in focus in association studies of schizophrenia before. In a recent study, this synonymous polymorphism was reported to be associated with schizophrenia in a South Indian population using a sample size of 101 individuals [12]. Interestingly, this study reported significantly higher association with H313H TT genotype in a recessive model which is in contrast to our observation on association of TT genotype using a dominant model as we observed higher H313H T allele frequency in our study population. This discrepancy could be due to sample size and population structure of the sampled population in a genetically diverse South Indian states. We further observed that the DRD2 Cys311 allele frequency (0.11 in patients and 0.10 in controls) was high in our South Indian population when compared with different world populations. The Cys311 allele frequency has been reported to range from 0.01 to 0.05 in Europeans and 0.00 to 0.06 in Asian population while our Cys311 allele frequency was comparable to North American Pima Indian population [19,35]. Arinami *et al*. [9] reported a positive association of the Cys311 allele with schizophrenia. But most of the later studies failed to support the argument that Cys311 allele of DRD2 poses a genetic risk factor for schizophrenia [14,20,36-42]. Meta-analyses of case control studies with DRD2 S311C provides strong evidence that Cys311 allele of DRD2 influences susceptibility to schizophrenia and that the risk associated with this polymorphism is actually greater than either HTR2A or DRD3 [19]. However, in the present study higher Cys311 allele frequency, does not support the association of Cys311 allele or genotype with schizophrenia but suggest for an ethnic bias in South Indian population. Golimbet et al. [43] suggested that TaqI A2A2 genotype may be a potential genetic factor for susceptibility to schizophrenia. Higher frequency of TaqI A2A2 genotype in our patient and control population was observed but no positive association with the disease could be established. However, in haplotypic combinations Taq1 A2 and H313H T alleles were significantly associated with the disease. This suggests that predisposition of an allele for any disease needs to be understood in terms of its haplotypic associations.

### Therapeutic response of DRD2 variants

Ethnic disparities in the prescription of antipsychotic drugs and their response in schizophrenic patients have been reported extensively [44-46]. Effective known treatments for schizophrenia are those that principally antagonize the DRD2 subtype of dopamine receptor [47]. All known antipsychotic drugs have potent affinities for the DRD2 receptor. Functional brain imaging studies have suggested DRD2 receptor binding by antipsychotic agents may be necessary and sufficient for antipsychotic efficacy [13]. Therefore, the DRD2 receptor gene becomes an obvious candidate for pharmacogenetic studies of antipsychotic drug response in schizophrenia. But not much of information is available on therapeutic response of antipsychotics in relation to DRD2 variants in schizophrenia. Differences in allelic or genotypic associations were observed with treatment response, in diverse ethnic groups. Recently DRD2 Taq1A1 allele has been correlated with favorable antipsychotic response [48]. Functionally A1 allele has been suggested to diminish dopaminergic activity in the CNS viz. prolonged p300 latency, reduced visuo-spatial function and reduced brain glucose metabolism [49]. Earlier reports also suggested that DRD2 Taq1A2A2 genotype individuals display poorer clinical response than their heterozygous counterparts [50] and this distinction was achieved within two weeks of treatment [51]. Our study did not find any difference in allele and genotype frequencies of Taq1A in responder and non responder groups. Hwang et al. [33] has reported that in Taq1B polymorphism, Taq1 B2 allele is associated with response to clozapine in African-American sample but not in Caucasian samples. This too could not be replicated in our sample, when allele and genotype associations were compared. Hwang et al. [33] has studied Taq1D polymorphism in Caucasian as well as African – American population, but did not find any significant observation for response to clozapine. In contrast, our study found significant association of D1D1 genotype in responders when compared to nonresponder group (p = 0.023) in a recessive model. Recently another DRD2 S311C variant has also been shown to have general treatment response to several atypical antipsychotics [52] which could not be confirmed in our study. Interestingly, the H313H TT genotype which was strongly associated with the disease was also found to be associated with good response (p = 0.0490). At this point it is difficult to interpret the functional significance for this tendency but it can be hypothesized that its linkage with other polymorphisms might be vital in altering the therapeutic response. In the present study we found that, the Taq1B, Taq1D, S311C and H313H were mainly associated with response, where B2 and D1 were associated with good response while Cys 311 and H313H C were associated with poor response. Hwang et al. [33] reported that among DRD2 SNPs, Taq1A, Taq1B and rs 1125394 could be used as markers for clozapine response in African-American patients while this does not hold true for Caucasian. Discrepancies between different studies are largely due to methods of evaluating response. It is difficult to generalize the response of single drug with multiple drugs, variable time intervals for evaluating response and classifying responders and non responders with different threshold for response. Besides the response scores are overall generalized scores. Therefore, in order to address this discrepancy, it would be ideal to understand the disease in terms of its presentation of symptoms in a particular ethnic group.

### Response of symptoms in general and in association to DRD2 variants

Dopamine receptors are involved in various responses such as motor control, neuroendocrine regulation, cognition, emotion and drug abuse that are critically impaired or associated with schizophrenia. In schizophrenia, it is not only ethnicity but various socio-cultural factors can also influence a particular symptom. In the present study, these socio-cultural factors are addressed by understanding the presentation of symptoms in the study population and subsequently evaluating the symptom and treatment response in relation to its genotype. We observed that 60% of the patient population presented certain positive symptoms such as suspiciousness, hallucinations, hostility and bizarre behavior and certain negative symptoms such as self neglect, blunted effect, emotional withdrawal and uncooperativeness. Cognitive symptom such as unusual thought content was also observed to be highly represented in the patient population. Same set of symptoms were also found to be chronic in presentation when severity of the symptoms were assessed, however, the severity of the presentation varied. Assessment of symptoms based on one year follow up of antipsychotic medication indicated that the presentation of symptoms varied in responder and non responder groups. In responder group the representation of bizarre behaviour, self neglect, hostility and unusual thought content reduced drastically while a reverse trend was observed in the non-responder group. However, aggressive symptom such as hostility reduced in its representation in both the group, while blunted effect was observed to have been minimally reduced or remained unchanged in its representation. Even the severity scores of various symptoms in the responder group were observed to decrease drastically when compared to their non-responder group patients. Similar variations in the presentation of symptoms have been reported earlier. Patel et al. [53] reported that Caucasian patients have more severe and persistent mood symptoms when compared to African-American and Hispanic ethnic groups and also suggested that it may alter the response to atypical antipsychotic risperidone in schizo-affective disorders. Emsley et al. [54] has observed that patients of Asian and Hispanic origin have better response to antipsychotics at a low dose than Caucasian counter parts. In a two-year follow up study involving eight countries, 50% of Indian schizophrenic patients were reported to have recovered in India and 58% in Nigeria, but only 8% were reported as recovered in Denmark [55]. Thus it can be suggested that ethnicity may play a role in presentation of symptoms and treatment response but whether there is any relation between dopaminergic activities based on genotype to phenotype correlations is still not clear. Therefore relating the phenotype to its corresponding genotype seems to be crucial in understanding the heterogeneity of the disease.

A strong association of TaqI B1B1 genotype with severe hallucination and TaqI A1A1 and A1A2 genotypes with severe self neglect symptoms was observed. H313H TC and TT genotypes were strongly associated with severe self neglect and uncooperativeness respectively in schizophrenic patients. Thus it can be suggested that in DRD2 TaqI A the A1 allele and in H313H the T allele are indicative of severe psychopathological symptoms. While assessing the relationship of genotype to a specific phenotype with response to medication, we observed better response with TaqI D1D1 genotype for suspiciousness, hallucination and bizarre behaviour while their alternate homozygous genotypes are indicative of poor response to medication for these symptoms. Heterozygous H313H TC genotype individuals show poor response to medication for hallucination symptom. However, among negative symptoms homozygous H313H TT genotype was observed to be associated with reduced impact of medication on uncooperative behaviour. DRD2 TaqI A2A2 and B2B2 genotype was observed to be associated with improvement of self neglect symptom. Interestingly, the genotypes that were strongly associated with chronic presentation of symptoms were not associated with good response. However, H313H TT and TC genotypes that were found to be associated with severe negative symptoms such as self neglect and uncooperativeness were also found to be associated with poor response to medication. Since the present study is exploratory in nature it is difficult to assign any specific functional role of these genotypes to any specific symptom, as these symptoms are also likely to be associated with multiple genes. In conclusion it can be suggested that in South Indian population H313H and TaqIA polymorphism are the most critical polymorphism that are involved in predisposition to disease, presentation of individual symptoms and in treatment response.

This presumably is the first exploratory study which attempts to understand the genotype phenotype relationship in a disease to the level of its individual symptoms. The symptoms of schizophrenia are so complex in nature that it needs adequate time and various drugs for appropriate response. Therefore, the study was set to its natural background without controlling the antipsychotic medication till the one year follow-up period. Correction for multiple hypotheses was not tested as this would drastically reduce the resolution of individual observation with the available sample size. Therefore a larger study would resolve the genotype phenotype correlations to the level of individual symptoms more precisely. The present study suggests that the disparity in the management of schizophrenia should not only be interpreted from its ethnic perspective but also through its relationship to social and cultural events associated with genotype phenotype correlations in a specific ethnic community.

## Competing interests

The author(s) declare that they have no competing interests.

## Authors' contributions

NNV carried out the molecular genetic studies, did the statistical analyses and drafted the manuscript. SB, LKV, CN and LS participated in the sample collection and statistical analyses. CN and PA recruited patients and did the psychiatric ratings. MB conceived the study, and participated in its design and coordination and helped to draft the manuscript. All authors read and approved the final manuscript.

## Supplementary Material

Additional file 1 Statistical analysis of interaction of BPRS score with genotype. Percent Improvement in BPRS scores associated with genotype using Mann Whitney U test. Click here for file
