# Supplementary material for: Association of dopamine receptor polymorphisms with schizophrenia and antipsychotic response in a South Indian population
Source: Behav Brain Funct. 2007 Jul 25;3:34. doi: 10.1186/1744-9081-3-34 (PMC1947997; doi:10.1186/1744-9081-3-34)
Supplement: Additional file 1 — Statistical analysis of interaction of BPRS score with genotype. Percent Improvement in BPRS scores associated with genotype using Mann Whitney U test. [file 1744-9081-3-34-S1.doc]

**Additional file 1**: Percent Improvement in BPRS scores associated with genotype using Mann Whitney test.

Polymorphism Genotype Mean Rank U 2 Tailed

*P* Value

S311C GC x GG  GC  85.89 2542.0 0.83 GG  87.92 2426.0

His313His   CC x TC  CC  71.75 1333.5 0.04

TC  57.76 2110.5

His313His  CC x  TT  CC  51.23 1061.5 0.59

TT  47.30 1248.5

His313His   TC x  TT    TC  64.10 2656.5 0.08

TT  76.30 1853.5

Taq1A  A1A1 x A1A2  A1A1  49.69 550.0 0.98

A1A2  49.47 555.0

Taq1A A1A1 xA2A2  A1A1  39.04 623.5 0.25

A2A2  48.29 416.5

Taq1A  A1A2 x A2A2  A1A2  75.89 4004.5 0.04

A2A2  90.56 2795.5

Taq1D  D1D1 xD1D2  D1D1  81.80 634.0  0.006 D1D2  58.16 1426.

Taq1D   D1D1xD2D2  D1D1  48.45 341.0 0.01

D2D2  34.20 759.0

Taq1D   D1D2 xD2D2  D1D2  78.95 2889.5 0.01

D2D2  80.54 2775.5

Taq1B B1B1 x B1B2 B1B1  44.71 254.0 0.80 B1B2  42.30 285.0

Taq1B B1B1 xB2B2  B1B1  46.07 363.5 0.64

B2B2  51.37 294.5

Taq1B B1B2xB2B2  B1B2  79.46 4122.5 0.12 B2B2  91.36 3115.5
